# Prospective observational study to assess hand skin condition after application of alcohol-based hand rubs solutions

**DOI:** 10.1186/1753-6561-5-S6-P272

**Published:** 2011-06-29

**Authors:** D Ahmed Lecheheb, L Cunat, P Hartemann, A Hautemaniere

**Affiliations:** 1 Department of Public Health and Environment, Medicine University of Nancy , Vandoeuvre les Nancy, Nancy, France; 2 RHEM 4369 Relations Environments Micro-Organisms, Medicine University of Nancy, Nancy, France; 3 Service d’Hygiène Hospitalière, University Hospital of Nancy, Vandoeuvre les Nancy, France

## Introduction / objectives

Deterioration in skin condition leads to reduced barrier function, changes in skin flora, and increased bacterial shedding. Thus, poor skin condition can increase the risk of infection transmission. The aim of this study was to assess the hand skin condition and dermal tolerance, among Health Care Workers (HCWs) after application of Alcohol-based hand rub solution (ABHRS).

## Methods

231 HCWs were included, 33.8% nurses, 22.1% nurse assistants and 14.7% hospital cleaners. (Mean age 40 years). The stratum corneum hydration, superficial sebum content and surface pH of the skin were measured on the back and the palm of the dominant hand before and after one application of ABHRS. *t*-test for paired samples was used to compare measurements of skin condition before versus after rub. A self-assessment questionnaire was administered in order to collect information about HCWs, skin problems and tolerance of ABHRS during daily use.

## Results

The skin hydration increased significantly after application of ABHRS for the two sites of measurements (*p* = .0001). The mean of pH values did not change significantly on the back of hand, but there were a significant changes for the palm (-0.069 ± 0.41, *p* = .012).Superficial sebum content decreased significantly after rub on the palm (-0.53 ± 1.56, *p* = .0001), but no significant difference was observed for the back (*p* = .076). 73% of HCWs reported an excellent or good skin tolerance of ABHRS.

## Conclusion

ABHRS are well tolerated and do not dry the skin; pH and superficial sebum decreased slightly, but not affected the skin barrier function. Values were also in the physiological range.

## Disclosure of interest

None declared.

